# Energy-Efficiency Analysis of a Distributed Queuing Medium Access Control Protocol for Biomedical Wireless Sensor Networks in Saturation Conditions

**DOI:** 10.3390/s110201277

**Published:** 2011-01-25

**Authors:** Begonya Otal, Luis Alonso, Christos Verikoukis

**Affiliations:** 1 Institute of Biomedical Research August Pi Sunyer (IDIBAPS), Barcelona, Spain; E-Mail: botal@clinic.ub.es; 2 Technical University of Catalonia (UPC), Castelldefels, Spain; E-Mail: luisg@tsc.upc.edu; 3 Telecommunications Technological Centre of Catalonia (CTTC), Castelldefels, Spain

**Keywords:** body sensor networks, distributed queuing, energy-efficiency, medium access control, power consumption

## Abstract

The aging population and the high quality of life expectations in our society lead to the need of more efficient and affordable healthcare solutions. For this reason, this paper aims for the optimization of Medium Access Control (MAC) protocols for biomedical wireless sensor networks or wireless Body Sensor Networks (BSNs). The hereby presented schemes always have in mind the efficient management of channel resources and the overall minimization of sensors’ energy consumption in order to prolong sensors’ battery life. The fact that the IEEE 802.15.4 MAC does not fully satisfy BSN requirements highlights the need for the design of new scalable MAC solutions, which guarantee low-power consumption to the maximum number of body sensors in high density areas (*i.e.*, in saturation conditions). In order to emphasize IEEE 802.15.4 MAC limitations, this article presents a detailed overview of this de facto standard for Wireless Sensor Networks (WSNs), which serves as a link for the introduction and initial description of our here proposed Distributed Queuing (DQ) MAC protocol for BSN scenarios. Within this framework, an extensive DQ MAC energy-consumption analysis in saturation conditions is presented to be able to evaluate its performance in relation to IEEE 802.5.4 MAC in highly dense BSNs. The obtained results show that the proposed scheme outperforms IEEE 802.15.4 MAC in average energy consumption per information bit, thus providing a better overall performance that scales appropriately to BSNs under high traffic conditions. These benefits are obtained by eliminating back-off periods and collisions in data packet transmissions, while minimizing the control overhead.

## 1. Introduction

The release of IEEE 802.15.4 for Low Rate Wireless Personal Area Networks (LR-WPAN) [1] represents a milestone in Wireless Sensor Networks (WSNs), and is the current standard of choice for most Body Sensor Networks (BSNs) studied scenarios. It targets low data rate, low power consumption and low cost wireless networking and offers device level wireless connectivity. It is expected to be used in a wide variety of embedded applications, including home automation, industrial sensing, environmental control and medical monitoring. In these applications, numerous embedded devices running on batteries are distributed in an area communicating via wireless radios. The key concern is thereby that of extremely low power consumption, since it is often infeasible to replace or recharge batteries for the devices on a regular basis.

Similar to all IEEE 802 wireless standards, the IEEE 802.15.4 standard standardizes only the physical (PHY) and medium access control (MAC) layers [1]. Here we concentrate however on MAC layer protocols, which play a significant role in determining the efficiency of wireless channel bandwidth sharing an energy cost of communication. In the IEEE 802.15.4 MAC layer, a central controller in a LR-WPAN, called the Personal Area Network (PAN) coordinator, builds the network in its personal operating space. The standard supports three topologies: star, peer-to-peer and cluster-tree. The star topology communication is established between devices and the PAN coordinator; in the peer-to-peer topology any device can communicate with each other device within its range; and in the cluster-tree topology, most devices can communicate with each other within the cluster, but only some of them may connect to the infrastructure. The standard identifies two channel access mechanisms:

- Beacon-enabled networks use a slotted Carrier Sense Multiple Access mechanism with Collision Avoidance (CSMA/CA), and the slot boundaries of each device are aligned with the slot boundaries of the PAN coordinator. The communication is then controlled by the PAN coordinator, which transmits regular beacons for device synchronization and network association control. The PAN coordinator defines the start and the end of the superframe by transmitting a periodic beacon. The length of the beacon period and hence the duty cycle of the system can be defined by the user between certain limits as specified in the standard [1]. There are 16 time slots in a superframe. Among them, there are at most seven Guaranteed Time Slots (GTS) that form the Contention Free Period (CFP), and the others are Contention Access Period (CAP). The advantage of this mode is that the coordinator can communicate at will with all nodes. The disadvantage is that nodes must wake up to receive the beacon.
- In non-beacon mode, a network node can send data to the coordinator at will, using a simpler unslotted CSMA/CA, if required. If the channel is idle, following a random back-off, the transmission is performed. If a busy channel is detected, the device shall wait for another random period before trying to access the channel again. To receive data from the coordinator the node must power up and poll the coordinator. To achieve the required node lifetime the polling frequency must be predetermined by power reserves and expected data quantity. The advantage of the non-beacon mode is that the node’s receiver does not have to regularly power-up to receive the beacon. The disadvantage is that the coordinator cannot communicate at will with the node but must wait to be invited by the node to communicate.

**Figure 1.**
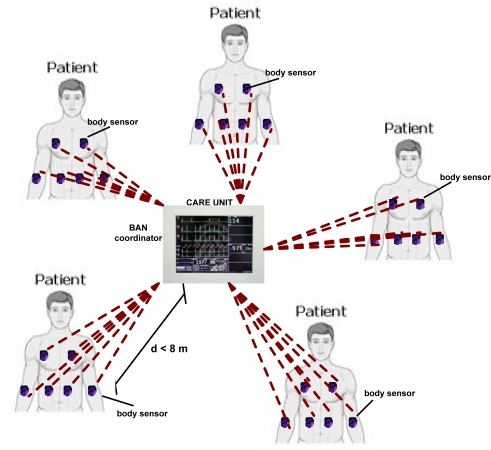
A star-based BSN in a potential medical scenario.

We focus here on single-hop beacon-enabled star-based BSNs, where a BAN coordinator is elected. That is, from now on we refer to a BAN instead of a PAN, while using either the IEEE 802.15.4 MAC or our newly proposed Distributed Queuing (DQ) MAC. In a ward BSN as portrayed in Figure 1, the BAN coordinator can be, for example, a bedside monitoring system, with several ward-patients wearing body sensors. Single-hop communication from body sensors to BAN coordinator (uplink), from BAN coordinator to body sensors (downlink), or even from body sensor to body sensor (*ad hoc*) is possible. In the following, we model the uplink communication, which occurs more often than downlink or *ad hoc* communication for regular patient monitoring BSNs in hospital environments (see Figure 1). That is, we consider hereby that a single-hop star-based (uplink) setting is more directly suitable than an *ad hoc* or multi-hop setting for current BSNs, since we think of healthcare scenarios in which most wearable medical body sensors report to a single medical care unit in the nearby with more energy-resources (see next Section 1.1). Further, it might be pointed out that a multi-hop scenario may be too energy-hungry for some specific body sensors (e.g., the ones close to the care unit) and thus jeopardize their own medical data transmissions. It is therefore assumed that the single-hop star-based topology is the most energy-efficient, because this does not put body sensor’s own medical data transmission at risk, avoiding unnecessary battery replacements.

**Figure 2.**
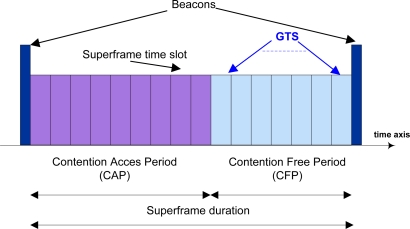
IEEE 802.15.4 MAC superframe structure in beacon-enabled mode (active).

In a IEEE 802.15.4 star-based BSN, the beacon-enabled mode appears to allow for the greatest energy efficiency. Indeed, it allows the transceiver to be completely switched off up to 15/16 of the time when nothing is transmitted/received, while still allowing the transceiver to be associated to the network and able to transmit or receive a packet at any time [2]. The beacon mode introduces the so-called superframe structure (Figure 2).

As previously mentioned, the superframe structure starts with the beacon, which is a small synchronization packet sent by the BAN coordinator, carrying service information for the BSN maintenance and notifying body sensors about pending data in the downlink. The inter-beacon period is partially or entirely occupied by the superframe, divided into 16 slots. The number of slots at the tail of the superframe may be used as GTS, *i.e.*, they can be dedicated to specific body sensors with no contention (see Figure 2, CFP). This functionality targets very low latency applications, but does not scale properly to highly dense BSNs (*i.e.*, saturation conditions), since the number of dedicated slots would not be sufficient to accommodate more than seven body sensors at a time. In such conditions, it is better to use the contention access mode, where the sparse data is statistically multiplexed. In the contention access period, distributed channel accesses in the uplink are coordinated by a slotted CSMA/CA mechanism, while indirect transmission is used in the downlink. As we will see later, the CSMA/CA mechanism has a significant impact on the overall energy and performance of the uplink. According to the slotted CSMA/CA algorithm in [2], a node must sense the channel free at least twice before being able to transmit, this corresponds to the decrement of the so-called contention windows. The first sense must be delayed by a random delay chosen between 0 and 2^BE–1^, where BE is the back-off exponent. This randomness serves to reduce the probability of collision when two nodes simultaneously sense the channel, assess it free and decide to transmit at the same time. When the channel is sensed busy, transmission may not occur and the next channel sense is scheduled after a new random delay computed with an incremented back-off exponent. If the latter has been incremented twice and the channel is not sensed to be free, a transmission failure is notified and the procedure is aborted. When a packet collides or is corrupted, it can be retransmitted after a new contention procedure. The contention procedure starts immediately after the end of the beacon transmission (see Figure 2, CAP). All channel senses or transmissions must be aligned with the CSMA slot boundaries that are separated by a fixed period.

### 1.1. IEEE 802.15.4 Suitability for BSNs in Medical Applications

From the traffic point of view, we can broadly group the BSN medical applications into three categories: real time low data rate, best-effort low data rate, and real time high data rate. The first category includes EEG (Electroencephalography), ECG (Electrocardiogram), blood analysis, *etc*. The second category consists of supervisors, control and alarms, *etc*. The third category covers EMG (Electromyography) and endoscope. All of these medical signals have very strict requirements in terms of accuracy, reliability and latency, since some of them are life critical. ECG is an electrical recording of voltage in the heart in the form of continuous strip graph, which has a prime function in screening and diagnosis of cardiovascular diseases [3].

In healthcare scenarios, where different patients are treated simultaneously, the traffic generated by the body sensors deployed in a hospital room can also be classified in two different types: periodic and aperiodic. Periodic traffic contains the routine check-up values for the patients and physical status of the room. Usually, these values do not have strict timeliness requirements. Therefore, normal contention access method (CSMA/CA) may be used for such traffic. However, the aperiodic traffic, which is generated on the basis of some unexpected event, occurred with the patients or within the room, is very critical and needs guaranteed access to the channel and bandwidth as they must report before a worse case situation happens (*i.e.*, strict latency requirements). Examples for such traffic are; (i) a dramatically increase/decrease in the blood pressure; or, (ii) a heart-attack to the patient; or, (iii) an unexpected temperature change in the room. GTS services can be used for such traffic.

It looks straightforward to implement IEEE 802.15.4 in such scenarios, but following limitations of the protocol must be figured out before attempting this. The first and foremost problem with the current GTS allocation is the bandwidth under utilization. Most of the time, a device uses only a small portion of the allocated GTS slots, the major portion remains unused resulting in an empty hole in the CFP, which represents a waste of the already scarce radio resources. As shown in Figure 2, the protocol explicitly supports only seven GTS allocations. In the medical field, where one illness usually boost-ups other illnesses, many devices should be able to reach the coordinator via such guaranteed services. Besides, the current protocol only supports first come first serve based GTS allocation and does not take into account the traffic specification, delay requirements, and the energy resources. In medical scenarios, many critical events may occur at a time, and some of them are more critical and need most urgent response. With the current protocol, the device can request for all seven GTS slots, even if it is not really needed. Such unbalanced slot distribution can block other needful devices to take such timeliness advantages. Moreover, the protocol uses GTS expiration on the basis of some constant factors and the assigned GTS slots are broadcasted for the constant number of times in the superframes. Such restrictions also cause unnecessary energy consumption and CFP slots blockage for longer time. Even if CFP is not present in the superframe, the beacons transmitted by the coordinator always use unnecessarily one byte of the CFP, resulting in certain energy inefficiency. Last but not least, the current IEEE 802.15.4 superframe structure contains a constant size CAP. For most urgent scenarios, we may need flexible size CAP rather than the fixed one.

### 1.2. IEEE 802.15.4 Limitations on Energy Consumption

The performance evaluation study in [4] reveals some of the key throughput-energy-delay tradeoffs in the IEEE 802.15.4 MAC. The authors provide an analysis comparing the energy costs of beacon and non-beacon modes for synchronization, showing that the optimum choice depends upon the combination of duty cycles and data rates. In [5], a Markov chain model of the IEEE 802.15.4 MAC is proposed, where each state is based on the counter values as the 802.11 model in [6]. Both models describe the behavior of the protocols using the probability that the device is in the channel accessing states. However, in the IEEE 802.15.4 MAC, this probability is not suitable for describing the behaviors because the channel sensing should be performed twice before entering accessing states. In [7], Park *et al*. propose a new Markov chain model of IEEE 802.15.4 and analyze the throughput and energy consumption in saturation conditions. The proposed model utilizes the probability of a device in the channel sensing states instead of in the channel accessing states. A similar approach for evaluating the performance of slotted IEEE 802.15.4 was followed by Pollin *et al*. in [8]. The model and analysis are similar in form to Bianchi’s [6], but here the key approximation in their model is the independence of the carrier sensing probability, which determines when nodes become active to listen to the channel.

Both analytical models in [7] and 8] show how the gross saturation throughput, expressed as the number of occupied slots for successful packet transmissions of size L (ignoring protocol over-head), drastically decreases as the number of sensors in the network increases. Energy consumption per information bit is also obtained through both of these models, presenting their worst results for a high number of nodes (e.g., 20–40 nodes). Information bit is here defined as the payload bit in the data packet, *i.e.*, non-control bit.

It is therefore deduced that the IEEE 802.15.4 MAC may jeopardize the deployment and scalability of BSNs, not only in terms of throughput, but especially in terms of energy consumption. Thus, the IEEE 802.15.4 MAC performance should be improved, targeting at low power consumption MAC protocols that scale up within BSN scenarios, while fulfilling medical application requirements. We observe that Distributed Queuing (DQ) MAC protocols [9–11] present a large number of advantages with respect to CSMA-based wireless communications systems. Therefore, we further analyze and optimize this DQ MAC scheme in order to prove its performance within BSN scenarios, and to improve the radio channel utilization taking the 802.14.5 MAC standard as a reference.

Please note that the introduction of energy-aware radio activation polices into a DQ MAC mechanism, as also an energy-consumption analysis in non-saturation conditions was already introduced in [12]. An optimization design and evaluation of the here characterized DQ MAC protocol in terms of quality-of-service was presented in [13] under different healthcare scenarios. The aim of [13] was to study a novel cross-layer fuzzy-rule based scheduling algorithm, which allowed packet transmissions to be scheduled taking into account the channel quality among body sensors, each sensor specific medical constraints and their residual battery lifetime.

In the next sections, we further analyze the potential benefits of DQ MAC in terms of energy efficiency per information bit in BSNs under saturation conditions. That is, analytical results of average energy consumption per information bit are presented in order to get a measure of the obtainable benefits of using this DQ MAC proposal compared to IEEE 802.15.4 MAC in extensive healthcare scenarios with a raising number of body sensors in the same area (*i.e.*, high density area).

## 2. An Energy-Saving DQ MAC Protocol for BSNs

The Distributed Queuing Random Access Protocol (DQRAP) is a random access protocol based on a queuing system shared among nodes. It was proposed for the first time in 1992 by Xu and Campbell [9,10]. Starting from a previous protocol called DQDB (Distributed Queuing Dual Bus), they developed the DQRAP protocol for a TDMA environment proposing an analytical model and showing, by means of computer simulations, how the protocol approaches the performance of the theoretical optimum system M/M/1. DQRAP divides the TDMA slot into a “reservation subslot” or “control subslot”, that is further divided into access minislots, and a data subslot. The basic idea of DQRAP is to concentrate user accesses in the control subslot, while the data subslot is devoted to collision free data transmission. The DQRAP provides a collision resolution tree algorithm that results stable for every traffic load even over the system transmission capacity. One of the most interesting features of the DQRAP protocol is its capacity to behave like an ALOHA-type protocol for light traffic load and to smoothly switch to a reservation system as the traffic load increases, reducing automatically collisions. Moreover, it must be taken into account that the protocol is fair as all nodes get, on average, the same service from the system. An interesting property of the DQRAP comes from the distributed queue adoption. Nodes can estimate the system load simply considering the number of busy positions in each queue. The load estimation is important information in a network environment.

In 2003, based on their previous research works, Alonso *et al*. [11] presented the Distributed Queuing Collision Avoidance (DQCA), which is a distributed high-performance medium access protocol designed for WLAN environments. The protocol behaves as a random access mechanism under low traffic conditions and switches smoothly and automatically to a reservation scheme when traffic load grows. DQCA has the following main features:

▪ It eliminates back-off periods and collisions in data packet transmissions.
▪ It performs independently of the number of stations transmitting in the system.
▪ It does not suffer from instability under all traffic conditions like slotted Aloha and keeps maximum achieved transmission rate if arrival rate keeps growing.

Crucial success of BSNs is the availability of small, lightweight, low-cost body sensors. Even more important for medical applications, the body sensors must consume low power to eliminate frequent battery replacement, at the same time that high reliability is guaranteed [13]. The preceding IEEE 802.15.4 MAC has all the aforementioned weaknesses when employed in BSNs for medical applications. However, the DQ MAC family introduces a range of advantages that we would like to further analyze from the energy-consumption perspective under BSNs in saturation conditions (*i.e.*, high density area).

### 2.1. Energy-Saving DQ MAC Superframe

**Figure 3.**
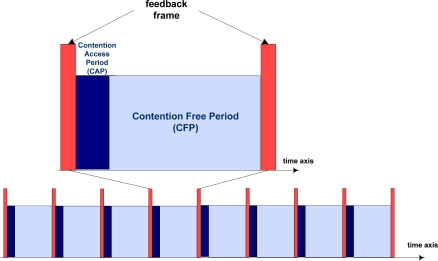
General superframe structure of a DQ MAC scheme.

The proposed DQ MAC mechanism is a distributed always-stable high performance protocol, which behaves as a random access mechanism for low traffic load and switches smoothly and automatically to a reservation scheme when traffic load grows. The key feature of the proposed protocol is that it eliminates collisions and back-off periods in data packet transmissions. Figure 3 portrays the general superframe format of a DQ MAC mechanism. In our proposal for a star-based wireless BSN, the complete DQ MAC superframe structure comprises two differential parts:

- From body sensors to BAN coordinator (uplink). This is through a CAP, specifically for body sensors’ access requests, and a CFP, exclusively for collision-free data transmissions;
- From BAN coordinator to body sensors (downlink). The BAN coordinator uses the feedback frame in order to acknowledge the previous data transmission and to broadcast control information to all body sensors in the BSN, so that they can follow independently the protocol rules (see [9–11]).

**Figure 4.**
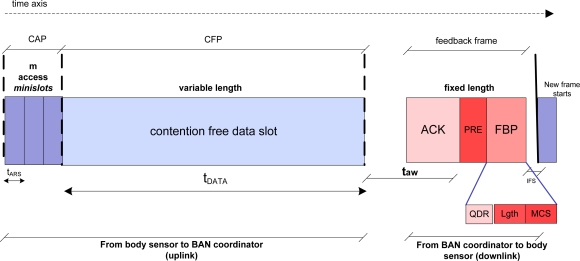
Energy-saving DQ MAC Superframe for BSNs.

Figure 4 depicts in more detail the superframe format of our adapted energy-saving DQ MAC first proposal for BSNs in healthcare environments.

#### DQ MAC Contention Access Period (CAP)

The CAP is further divided into *m* access minislots. Within these access minislots, Access Requests Sequences (ARS) of duration *t_ARS_* are sent to gather a position within the CFP. ARS are the minimum signal required for the BAN coordinator to detect channel access. That means, the PHY level only needs to detect three different states (empty, success, collision), but in principle no information bits are required [9,10], though it is implementation dependant.

#### DQ MAC Contention Free Period (CFP)

Immediately after the CAP, the CFP allows contention free data transmissions within the contention free data slot of variable duration *t_DATA_* or variable length (*i.e.*, payload length) (see Figure 4).

#### DQ MAC Feedback Frame

Following the contention free data slot, we define here *t_aw_* as the maximum time to wait for an acknowledgement (ACK) of duration *t_ACK_*, as in IEEE 802.15.4 [1]. Bear in mind that the DQ MAC superframe is bounded by the hereby named Feedback Packet (FBP), of fixed duration *t_FBP_*, contained in the depicted feedback frame (see Figure 3). Similar to IEEE 802.15.4 MAC superframe format, one of the main uses of the FBP is to synchronize the attached body sensors to the BAN coordinator. The FBP always contains relevant MAC control information, which is essential for the right functioning of all the attached body sensors to the BSN using the DQ MAC protocol. In our energy-saving DQ MAC superframe proposal, the FBP is preceded by a novel synchronization preamble (PRE), which follows immediately after *t_aw_* elapses. The functionality of PRE is to enable power management solutions and energy-aware radio activation policies among the different time intervals in order to prolong body sensors’ battery lifetime. At the end of the DQ MAC superframe, an Inter Frame Space (IFS) is added to allow the MAC layer to process the data received from the PHY layer.

All in all, the main differences of this new energy-saving DQ MAC superframe format with respect to the previous DQ MAC ones are the following:

1. A preamble (PRE) is newly introduced within the broadcasted feedback frame, concretely between the ACK and the FPB, to enable synchronization after power-sleep modus (*i.e.*, either idle or shutdown, see [2]). The intuitive reasoning is the following: (i) The feedback frame is an aggregation of an ACK and the FBP in order to save PHY header overhead and therefore energy-consumption at reception. That is, the ACK is essential only to the body sensor, who transmitted in the previous contention free data slot. Hence, body sensors can prolong their power-sleep modus until the immediate reception of the FBP. (ii) The precise position of the PRE between the ACK and the FBP is mainly due to scalability in terms of energy-efficiency. This means that in a future system design or downlink multicast applications, several ACKs or any other type of info, may be aggregated just before the preamble—PRE. Body sensors within the DQ MAC system not being addressed in this multicast/aggregated communication shall only receive the FBP. That is the reason why a preamble is suitable in this explicit position.
2. FBP is here of fixed length (*i.e.*, independent of the number of sensors in the BSN) and contains two brand new fields for specific energy-saving purposes; the Modulation and Coding Scheme (MCS) and Length of the packet transmitting in the next contention free data slot. This facilitates independent energy-aware radio activation policies; so that body sensors can calculate the time they can remain in power-sleep modus. Further, the MCS field is also thought for future multi-rate medical applications in BSNs (*i.e.*, scalability).

Note that the FBP always contains a specific field named QDR (Queuing Discipline Rules), which contains the updating information regarding the aforementioned ARS. That is, the QDR field contains the state of each of the access minislots, which can be empty, success, or collision. Two bits are necessary to encode the state of each access minislot, so 2 · *m* is the total number of bits devoted to the QDR field. (see [9,10]). Additionally, there is the possibility to transmit data packets of variable length (*t_DATA_*), using the same frame structure, at the same time that energy-saving benefits are maintained.

### 2.2. DQ MAC Data Transaction in a Star-Based BSN

Three types of direct data transfer transactions may exist in a stand-alone BSN. The first one is the data transfer from any body sensor to the BAN coordinator. The second type of transaction is the data transfer from the BAN coordinator to body sensors. The third transaction is the data transfer between two peer body sensors. In a star-based topology, only two of these transactions are used, because data may be exchanged only between the BAN coordinator and a body sensor. Hereby we choose to model and further study the single-hop star-based topology, because;

- it is the most feasible topology in current BSN medical applications scenarios (see [3]).
- it is the most suitable topology using DQ MAC. That is to say that a DQ MAC scheme already requires a centralized architecture [9], in spite of the distributed and independent behavior of all body sensors in the BSN.
- it is the most energy-efficient topology within direct short-range communication between body sensors and the BAN coordinator, taking single body sensor battery life-time into account.

Body sensors in medical applications are especially stringent in terms of power consumption. The replacement of batteries may cost more than the devices themselves, like for example in implantable devices. It is not only very cumbersome, but also practically impossible to replace the batteries in some applications at once. In a single-hop star-based BSN, body sensors save energy resources, because all data transmissions are directly to the BAN coordinator (within low-power range), which is elected considering its superiority in terms of power resources. That is, since no multi-hop data transmission is allowed, body sensors can manage their power consumption considering their own residual battery lifetime. That is the reason why a well-designed single-hop star-based BSN is more reasonable for short-range medical applications.

#### Data Transfer from a Body Sensor to a BAN Coordinator in a BSN

Our here referred DQ MAC protocol is based on two distributed queues. Note that for clarity reasons; only a brief explanation is included here. A more detailed description can be found in [9,10],

- the Collision Resolution Queue (CRQ); and
- the Data Transmission Queue (DTQ).

**Figure 5.**
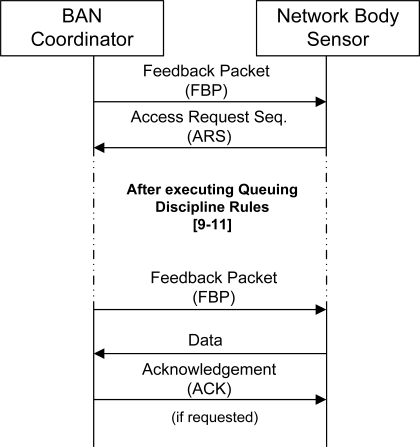
Communication between a body sensor and the BAN coordinator.

The CRQ is devoted to the collision resolution algorithm (for resolving ARS collisions) and the DTQ to collision-free data packet transmission scheduling. Both queues are simply represented by four integer numbers, recorded at every body sensor (*i.e.*, distributed queues), which correspond to the specific sensor position and the total number of sensors in each queue. Every body sensor updates these four numbers upon synchronization through the PRE and reception of the FBP. The information contained in the QDR field carries this specific information, seeFigure 4 [9]. Thereafter, every body sensor applies a set of rules to actualize its position in the CRQ and DTQ queues accordingly. At the appropriate time, a body sensor transmits whether a ARS within the CAP or its data packet within the CFP to the BAN coordinator. The BAN coordinator acknowledges the successful reception of the data packet by transmitting ACK. This sequence is summarized in more detail in the illustration of Figure 5.

**Figure 6.**
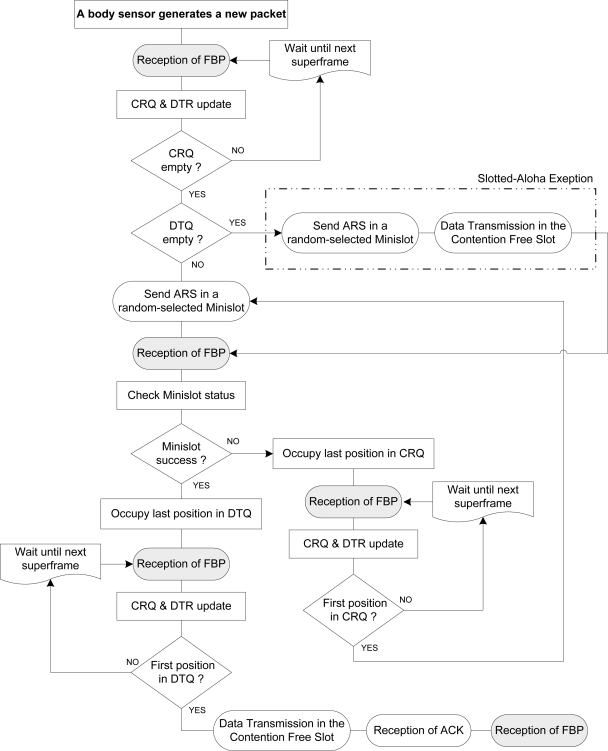
DQ MAC flow chart for energy-saving BSNs based on original DQRAP algorithm.

Further, Figure 6 specifically depicts a state-of-the-art DQ MAC data transfer from a body sensor to a BAN coordinator in a BSN, following DQ MAC protocol rules regarding CRQ and DTQ, after updating the state of the queues thanks to the information in the QDR field in FBP.

As aforementioned, a body sensor willing to transmit a packet must first synchronize with the BSN through the FBP broadcasted by the BAN coordinator to update the state of the system queues (CRQ & DTQ). Note that when both queues are empty, the protocol uses an exception of slotted-Aloha (see [9]). However, if CRQ is empty—but DTQ is not—the body sensor sends an ARS—randomly selecting one of the access minislots—to grant its access into DTQ. If its ARS collides with any of another body sensor in the selected access minislot, these body sensors involved therein occupy the same position in CRQ (following the order of the selected minislot position), and wait for a future frame to compete for a free access minislot again to grant its access into a DTQ exclusive position. New body sensors, with a packet to send, are not allowed to enter the system until CRQ is empty (*i.e.*, all current collisions are resolved). When a body sensor selects successfully a free access minislot (known at the reception of the FBP), it takes immediately a place in DTQ up. If DTQ is now empty, it may be in the first position of DTQ, thus transmitting directly in the next DQ MAC superframe data slot.

Note that a body sensor is just allowed to send an ARS in a random-selected minislot if and only if CRQ is empty. If CRQ is not empty, this body sensor has to wait until the next superframe and recheck again CRQ condition via the FBP. Therefore, the inherent behaviour of the protocol avoids new ARSs entering the system, if there are still old ARS to be resolved. That is, CRQ only has new entries, whenever it becomes empty.

## 3. Energy Consumption Analysis in Saturation Conditions

Like other different types of sensors, body sensors are limited in stored energy, computational capacity and memory. New MAC protocols and algorithms must be designed with special attention to these aspects and above all to their limited and sometimes non-renewable power storage. Significant power is consumed at a body sensor when it either transmits or receives a packet. Here we proceed to evaluate body sensors’ energy consumption in a star-based BSN using DQ MAC with respect to IEEE 802.15.4 MAC in saturation conditions (*i.e.,* highly dense BSNs), in which we suppose there are always sensors transmitting, and CRQ and DTQ are never empty (see Figure 6). That is, we assume a specific window size at a time for evaluating CRQ inner ARS resolution.

The DQ MAC energy consumption per information bit in saturation conditions *ɛ_bit_* (*i.e.*, efficiency) is here calculated as the average energy consumption to transmit one DQ MAC superframe *E_Superframe_* over the payload packet length in bits *L_bit_* of a superframe, considering all body sensors always have packets to send. Thus, the total energy consumption per information bit *ɛ_bit_* in saturation conditions for the whole superframe transmission can be expressed as follows:

(1) $${ɛ}_{\mathrm{bit}}=\frac{E_{\mathrm{Superframe}}}{L_{\mathrm{bit}}},$$

where *L_bit_* corresponds to the payload length in bits of the data packet transmitted in the collision-free data slot and *E_Superframe_* is defined as the energy consumption of the whole DQ MAC superframe from a body sensor perspective, taking uplink and downlink into consideration (see Figure 4).

To calculate the body sensor energy consumption for the whole DQ MAC superframe duration *E_Superframe_* (see Figure 4), let us first define the following:

- *E_ARS_*, and *E_DATA_*, as the energy consumption for ARS and data frame transmissions; and,
- *E_ACK_*, and *E_FBP_*, as the energy consumption for ACK and FBP frame receptions, respectively.

These energies can be obtained as:

(2) $$\begin{array}{l} E_{\mathrm{ARS}}={\mathrm{mt}}_{\mathrm{ARS}}P_{\mathrm{tx\_ARS}},\\ E_{\mathrm{DATA}}=t_{\mathrm{DATA}}P_{\mathrm{tx\_DATA}},\\ E_{\mathrm{ACK}}=t_{\mathrm{ACK}}P_{\mathrm{rx}},\\ E_{\mathrm{FBP}}=(t_{\mathrm{PRE}}+t_{\mathrm{FBP}})P_{\mathrm{rx}}. \end{array}$$

where *P_tx_DATA_* is the power consumption in transmission mode for a data frame, which according to Chipcon specifications [14] could differ from the power consumption of transmitting ARS (*P_tx_ARS_*), since in IEEE 802.15.4 MAC different transmission modes are allowed. Additionally, *P_rx_* is the power consumption in receiving mode. Further, Equation (2) also contains the average time the transceiver is in each of these four energy-consumption states corresponding to the DQ MAC superframe duration (see Figure 4), *t_ARS_* for ARS transmissions, *t_DATA_* for data transmission, *t_ACK_* for ACK reception, and, *t_PRE_*, *t_FBP_* for PRE synchronization and FBP reception, as aforementioned.

Thus, here *E_Superframe_* is computed as:

(3) $$E_{\mathrm{Superframe}}=E_{\mathrm{ARS}}+E_{\mathrm{DATA}}+E_{\mathrm{ACK}}+E_{\mathrm{FBP}}+\left(t_{\mathrm{aw}}-t_{\mathrm{ACK}}+t_{\mathrm{IFS}}\right)P_{\mathrm{idle}}.$$

Therefore, DQ MAC energy consumption efficiency *ɛ_bit_* is expressed in Joules per information bit (J/bit). Note that *P_idle_* corresponds to the state when a body sensor is neither transmitting nor receiving bits. That is to say, that a body sensor is not active or it is in idle mode. Typically, in general WSNs, the power consumed during receiving mode is larger than in transmitting mode [2]. The figures of the power consumptions utilized here in our case study are listed in Table 1 and based on Chipcon specifications [14]. For the sake of simplicity, in our calculations, we consider that both power consumptions in transmission mode (*i.e.*, *P_tx_DATA_* and *P_tx_ARS_*) have the same value, although they could differ from each other based on hardware specifications [14].

The reference scenario is defined by a set of parameters provided in Table 2, whose fields correspond to IEEE 802.15.4 MAC default values [1].

**Figure 7.**
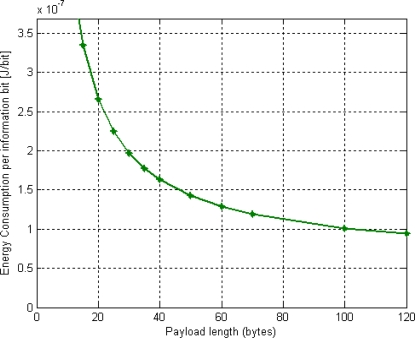
Energy consumption per information bit of DQ MAC protocol for BSNs.

Notice that the maximum Packet Service Data Unit (PSDU) the PHY layer shall be able to receive a packet in the IEEE 802.15.4 MAC standard is 127 bytes [1]. That is the reason why we study several lengths up to 118 bytes (aprox.120 bytes), assuming the minimum MAC overhead length of 9 bytes as indicated in Table 2. Further, in our DQ MAC protocol, the number of access minislots *m* is also 3, as in [9–11]. As previously said, the duration of the ARS (*t_ARS_*) could be reduced to a very small value (*i.e.*, between 2 μs and 10 μs), since no data information is needed to be carried through [9,10]. For our calculations though, we use 128 μs as ARS duration value in order to consider the worst-case scenario. This is equivalent to the duration of the Preamble sequence in IEEE 802.15.4 MAC and might be fare with a feasible hardware prototype implementation. Figure 7 characterizes the energy consumption per information bit of DQ MAC mechanism for BSNs derived from Equation (1) and the parameter values of Table 1 and Table 2. As expected, DQ MAC energy consumption per information bit decreases rapidly at increasing the payload length, due to the smaller relative overhead. DQ MAC mechanism maximum energy consumption is obtained for small-sized packets and reaches its maximum of 3.5 × 10^−7^ J/bit of the overall normalized energy consumption.

Following DQ MAC protocol specific rules, a body sensor is just allowed to send an ARS in a random-selected minislot, if and only if CRQ is empty. Unless CRQ is empty, this body sensor has to wait until the next superframe and recheck again CRQ condition via FBP (see Figure 6). Therefore, the inherent behaviour of the protocol avoids new ARSs entering the system, if there are still old ARS to be resolved. That is, CRQ only has new entries, whenever it becomes empty. In this particular scenario, we modelled a non-empty CRQ per window size, assuming on average *m* ARSs transmissions at a time in the process of CRQ becoming empty. That is in practice independently of which access minislot has been used, but in theory, we assume that there is no empty access minislot within that transmission. This assumption is valid in this specific conditions and scenario, since in [9], there is an example (for highly dense networks) where it can be seen that in stable conditions, with *m* = 3, after the third round trip, most devices have already entered DTQ. There, it is also proved the small difference between using *m* = 3 or *m* = 16 (a bigger number of minislots), showing that the delay to resolve collisions is minimal independently of the number of minislots. The specific rules of DQRAP (original DQ MAC) are explained in more detail in [9,10]. It is assumed that all blocked stations are supposed to transmit immediately whenever CRQ becomes empty, here synchronized via the FBP, and thereafter the protocol follows with the same behaviour. DQ MAC assures that the speed of contention resolution in the CRQ subsystem is faster than the speed of data transmission, thus guaranteeing that the CRQ subsystem will not block input traffic to the whole system [9,10].

### 

#### DQ MAC Energy Consumption Performance Evaluation

**Figure 8.**
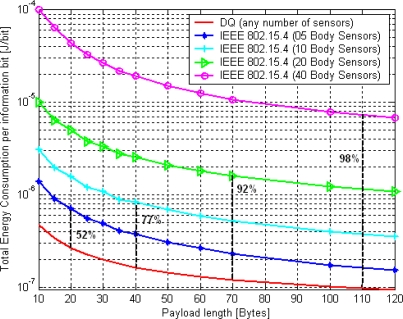
Achievable estimated energy consumption improvement per information bit of DQ MAC *vs*. IEEE 802.15.4 MAC.

To get example figures, a scenario with an increasing number of always-active body sensors in a star-based BSN in saturation has been selected. The next Figure 8 depicts the achievable estimated energy consumption improvement per information bit of DQ MAC mechanism *versus* IEEE 802.15.4 MAC protocol, derived from expressions (1) and [7], respectively. All IEEE 802.15.4 MAC curves are as a function of the payload length (in bytes) and the number of body sensors in the BSN. In saturation conditions, the IEEE 802.15.4 MAC shall deal with a certain level of data collisions, which steadily increases with the number of body sensors in the network. This results in a progressive reduction of the energy efficiency of the IEEE 802.15.4 MAC. In contrast, when evaluating DQ MAC protocol energy consumption per information bit in the same saturation conditions, we observed that DQ MAC energy efficiency is independent of the number of body sensors in the BSN, similar to the previous throughput analysis (see [9–11]) That is, because of the inherent behavior of DQ MAC of eliminating back-off periods and collisions in data transmissions by means of the distributed queuing system. This means, that in saturation conditions, DTQ is always non-empty, and collisions are gradually being resolved in the CRQ. As a result, no collisions are produced in the information data part of DQ MAC superframe (see Figure 4), and therefore no energy per information bit is wasted due to unwilling collisions. Like in the previous studied case, the reference scenario is defined by a set of parameters provided in Table 1 and Table 2, whose fields correspond to IEEE 802.15.4 MAC default values [1].

All data transmissions are supposed to be successful, except from the ones that fail anyway due to channel conditions, such as fading or Doppler Effect. Thus, although the collision resolution mechanism requires some energy consumption, the complete elimination of data collisions represents a remarkable enhancement in the overall network. That is:

▪ up to 98% improvement with respect to IEEE 802.15.4 MAC for a 40 body-sensor network size.
▪ up to 92% improvement with respect to IEEE 802.15.4 MAC for a 20 body-sensor network size.
▪ up to 77% improvement with respect to IEEE 802.15.4 MAC for a 10 body-sensor network size.
▪ up to 52% improvement with respect to IEEE 802.15.4 MAC for a 5 body-sensor network size.

## 4. Overall MAC Overhead Comparison in Saturation Conditions

**Figure 9.**
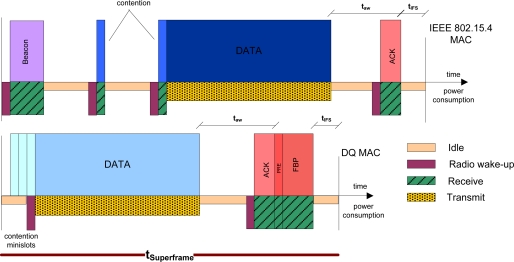
Energy consumption per information bit of DQ MAC protocol for BSNs.

Previously, we have compared the energy consumption per information bit of IEEE 802.15.4 MAC analytical model in [7] *versus* our proposed DQ MAC analytical scheme in saturation conditions. Next, we evaluate the overall MAC superframe overhead in terms of energy consumption based on the analysis in [2]. As sketched in both superframe structures of IEEE 802.15.4 MAC and DQ MAC in Figure 9, the IEEE 802.15.4 MAC introduces significant overhead, which has consequential impact on the overall energy consumption in saturation conditions. Please be aware that all depicted MAC fields follow IEEE 802.15.4 MAC values specified in Table 2 [1]. As aforementioned, to be fair in the comparison both IEEE 802.15.4 MAC and DQ MAC data packet lengths are the same, and the beacon and FBP fields have the same value respectively in their MAC superframes, *i.e.*, 11 bytes. In the following, we assume that body sensors attempt to transmit a single packet per superframe. Let us here assess the overall MAC overhead of the whole superframe structure in saturation conditions (*i.e.*, there is always a packet to be transmitted). To do so, in Figure 9 we clearly distinguished among the different power consumption states: transmit, receive and idle, like aforementioned in the previous studied scenario. In the IEEE 802.15.4 MAC superframe, a body sensor first listens to the beacon, after having preemptively turned on its radio in receive mode. After the beacon is received, the body sensor can enter in idle mode. The contention procedure requires at least two channel senses for CCA, which requires turning the receiver on. Within the CCAs, the receiver can return to the idle state. Once the channel is assessed clear twice, the transmission can start. If the packet is well received, a short ACK is fed back to the body sensor (in receiving mode) after a minimum time *t_aw_* when it is in idle state.

Figure 9 also shows our here proposed DQ MAC procedure, whose relative overhead compared to that of IEEE 802.15.4 is remarkably lower in saturation conditions. To compute the whole MAC overhead in DQ MAC in saturation conditions, we assume a body sensor is transmitting in the collision-free data slot, while its ARS has already been transmitted in a previous DQ MAC superframe. That is, in saturation conditions we compute the DQ MAC superframe energy-consumption per information bit.

**Figure 10.**
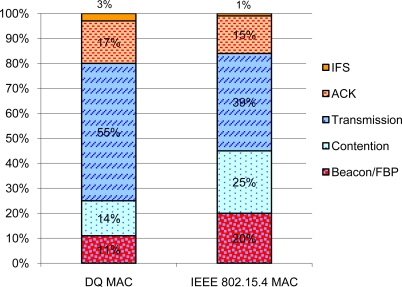
Breakdown of energy consumption.

To lower power consumption in future designs, it is valuable to know the power breakdown of both DQ MAC and IEEE 802.15.4 MAC superframe structures. Figure 10 presents the power breakdown between the different phases of the DQ MAC and IEEE 802.15.4 MAC considering their illustrations in Figure 9 and specified values in Table 2. We notice that the effective transmission in DQ MAC uses already more than 50% of the total energy consumption, which is an improvement with respect to IEEE 802.15.4, as analyzed in [2]. Less than 15% is spent during DQ MAC contention taking radio wake up polices into account. The ACK mechanism uses more than 15% of the energy, mainly because of the necessity of activating the receiver during the acknowledgement waiting-time *t_aw_*. For listening to the FBP, around 10% of the energy is used, compared to the 20% of energy spent in IEEE 802.15.4 in beacon mode. The rest is used for Inter-frame Space (IFS) purposes (*i.e.*, processing). Please note that all our analysis take radio wake up policies into account, for both DQ MAC and IEEE 802.15.4 MAC protocols.

## 5. Conclusions

In this article, we proposed a better conditioned energy-saving frame format of an enhanced Distributed Queuing Medium Access Protocol (DQ MAC) for Body Sensor Networks (BSN) in healthcare scenarios. Further, we have presented an analytical evaluation of this enhanced DQ MAC protocol for a star-based BSN until reaching saturation conditions (*i.e.*, high density area). It has been shown that our here proposed DQ MAC mechanism outperforms IEEE 802.15.4 MAC in terms of overall energy-consumption per information bit. All in all, we have shown that our proposed DQ MAC protocol represents a remarkable improvement of the overall network energy efficiency, which scales well for very dense BSNs and it is particularly suitable in medical scenarios.

## Figures and Tables

**Table 1.** IEEE 802.15.4 Transceiver Power Consumption (−25 dBm).

*P_tx_ARS_*	*P_tx_DATA_*	*P_rx_*	*P_idle_*
15 mW	15 mW	35.23 mW	712 μW

**Table 2.** IEEE 802.15.4 MAC & DQ MAC Parameter Values.

**Parameter**	**Value**	**Parameter**	**Value**
**PHY header**	6 bytes	**ACK**	11 bytes
**MAC header**	9 bytes	**Beacon**	11 bytes
**Data payload**	8 to 120 bytes	*t_aw_*	864 μs
**Data rate**	250 Kb/s	*t_IFS_*	192 μs
**DQ MAC**			
**Preamble**	4 bytes	*m*	3
**FBP**	11 bytes	*t_ARS_*	128 μs
